# *Staphylococcus aureus* Methicillin-Resistance Factor *fmtA* Is Regulated by the Global Regulator SarA

**DOI:** 10.1371/journal.pone.0043998

**Published:** 2012-08-30

**Authors:** Yinglu Zhao, Vidhu Verma, Antoaneta Belcheva, Atul Singh, Michael Fridman, Dasantila Golemi-Kotra

**Affiliations:** 1 Department of Biology, York University, Toronto, Ontario, Canada; 2 Department of Chemistry, York University, Toronto, Ontario, Canada; Institut Pasteur, France

## Abstract

*fmtA* encodes a low-affinity penicillin binding protein in *Staphylococcus aureus*. It is part of the core cell wall stimulon and is involved in methicillin resistance in *S. aureus*. Here, we report that the transcription factor, SarA, a pleiotropic regulator of virulence genes in *S. aureus,* regulates the expression of *fmtA*. *In vitro* binding studies with purified SarA revealed that it binds to specific sites within the 541-bp promoter region of *fmtA*. Mutation of a key residue of the regulatory activity of SarA (Arg90) abolished binding of SarA to the *fmtA* promoter, suggesting that SarA binds specifically to the *fmtA* promoter region. *In vivo* analysis of the *fmtA* promoter using a *lux operon* reporter fusion show high level expression following oxacillin induction, which was abrogated in a *sarA* mutant strain. These data suggest that SarA is essential for the induction of *fmtA* expression by cell wall-specific antibiotics. Further, *in vitro* transcription studies show that SarA enhances *fmtA* transcription and suggest that regulation of *fmtA* could be via a SigA-dependent mechanism. Overall, our results show that SarA plays a direct role in the regulation of *fmtA* expression via binding to the *fmtA* promoter.

## Introduction

*Staphylococcus aureus* is a versatile gram-positive pathogen capable of causing a wide range of diseases, ranging from superficial abscesses to pneumonia, endocarditis, and sepsis [1]. In the pre-antibiotic era, serious systemic staphylococcal infection was associated with 80% mortality [2]. When penicillin was introduced in 1944, over 94% of *S. aureus* isolates were susceptible. However by 1950, 50% of *S. aureus* isolates were penicillin-resistant, further demonstrating the remarkable ability of *S. aureus* to rapidly adapt to antibiotic pressure.

In 1960, outbreaks of virulent *S. aureus* that were resistant to penicillin occurred in many hospitals. These could be treated successfully with methicillin and other newly available penicillinase-stable penicillins. However, by 1961, two years after the introduction of methicillin, methicillin-resistant *Staphylococcus aureus* (MRSA) had emerged. Since then, a number of distinct MRSA strains have emerged and spread throughout the world [3]–[5]. MRSA, in addition to an intrinsic resistance to virtually all β-lactams, has an ability to accumulate and develop resistance to other, unrelated antibiotics [6]–[8]. Some variants of MRSA have developed resistance to glycopeptide antibiotics, including vancomycin, which is the sole antibiotic that can still be used to successfully treat most *S. aureus* [9].

*S. aureus* resistance to methcillin has been attributed to the acquisition of the *mecA* gene, which encodes a penicillin-binding protein (PBP) that is resistance to β-lactam inactivation, namely PBP2a; the four native PBPs of *S. aureus* are sensitive to β-lactams [6], [10], [11]. Despite the efficiency with which expression of PBP2a confers resistance to β-lactams, the PBP2a expression level does not correlate with high levels of methicillin resistance [12]–[15]. Other factors are suggested to play essential roles in the phenotypic expression of methicillin resistance [12]. Screening for methicillin-resistance factors has resulted in the identification of auxiliary genes including *fem, fmtA, llm*, sigma factor, *pbpD,* and *vraSR* [16]–[23]. These genes reside outside the *mecA* determinants [17] and have been shown to have direct or indirect roles in the biosynthesis or autolysis of peptidoglycan. The *fem* genes are involved in the biosynthesis of the peptide core of the peptidoglycan precursor subunit [24]–[26] and *pbpD*, which encodes PBP4 and is involved in the synthesis of highly cross-linked peptidoglycan [20].

Genome-based studies of the *S. aureus* response to cell wall-specific antibiotics identified *fmtA* as being part of the core cell wall stimulon [27]. The expression level of *fmtA* increases in the presence of cell wall inhibitors and when genes involved in biosynthesis of peptidoglycan are inactivated [27]–[31]. Insertions in *fmtA* reduce MICs of methicillin, cefoxitin and imipenem 8 to 16 fold for different MRSA strains. This effect is more pronounced in the presence of Triton X-100 [32], [33]. Insertions in *fmtA* also impair polysaccharide intercellular adhesion production and result in significantly reduced biofilm formation [34], [35]. Furthermore, the *fmtA* mutants exhibit reduced peptidoglycan cross-linking [32] and reduced attachment of wall teichoic acids to cell wall [34]. The *fmtA* gene product (FmtA) is capable of forming stable acyl-enzyme species with β-lactams, but the interaction is weak [36].

Upregulation of the *fmtA* expression by perturbation of peptidoglycan biosynthesis suggests presence of a regulatory mechanism capable of coordinating *fmtA* expression with cell wall biosynthesis. Here, we investigate the factors involved in regulation of *fmtA*.

In this study, we identified SarA as a transcription factor responsible for regulation of *fmtA*. SarA is a global regulator of *S. aureus* involved in the regulation of many virulence factors and was previously reported to be involved in methicillin resistance [37]. We report the DNA-binding sites of SarA in the *fmtA* promoter. The binding specificity of SarA to *fmtA* promoter region was probed by mutating a key functional residue of SarA (Arg90). In addition, *fmtA*-*lux* operon reporter constructs were used to investigate the regulation of *fmtA* expression *in vivo* in response to antibiotic stress. The activation of *fmtA* transcription by SarA was confirmed by *in vitro* run-off transcription assays. Together, our results show that SarA binds directly to the *fmtA* promoter and plays a direct role in the regulation of *fmtA* expression.

## Materials and Methods

### Growth Media and Chemicals

Chemicals were purchased from Sigma (Oakville, Canada) or Thermo-Fisher (Whitby, Canada), unless otherwise stated. Chromatography media and columns were purchased from GE Healthcare (Quebec, Canada). The growth media was purchased from Fisher. *Escherichia coli* Nova Blue and BL21(DE3) strains, as well as cloning and expression plasmids were purchased from EMD4 Biosciences (New Jersey, USA). Restriction enzymes were obtained from New England Biolabs Canada (Pickering, Canada) or Thermo-Fisher. All primers, including biotinylated primers, were purchased from Sigma (Oakville, Canada). The [γ-^32^P] ATP (3000 Ci/mmol) was purchased from Perkin Elmer LAS Canada Inc. (Toronto, Canada) or GE Healthcare (Quebec, Canada). The ProteoExtract All-in-One Trypsin Digestion Kit was purchased from EMD4 Bioscience.

### Preparation of Cell Extracts

*S. aureus* strain RN4220 (Cedarlane, Burlington, Canada) was grown to an optical density (OD) at 600 nm of approximately 0.9 in tryptic soy broth with or without oxacillin (1.2 µg/mL) and incubated for 1.5 h at 37°C. The cells were harvested at 4°C (11,000×*g*) and washed with cold TEG buffer (25 mM Tris-HCl, pH 8.0, and 5 mM EGTA) and re-suspended in TEG buffer. After two freeze-thaw cycles, cells were lysed with lysostaphin (0.3 mg/mL) followed by sonication. Lysates were centrifuged at 4°C (21,000×*g*) and 20% glycerol was added to the supernatants. The supernatants were dialyzed overnight in 1 L dialysis buffer (10 mM Tris-HCl pH 7.5, 1 mM EDTA, 1 mM DTT, 50 mM NaCl, and 20% (v/v) glycerol).

### Electro-mobility Shift Assay (EMSA)

**Figure 1:**
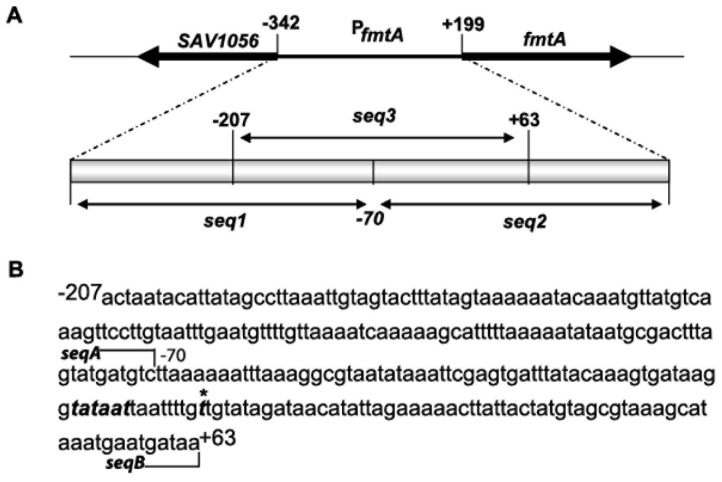
The *fmtA* promoter region in the *S. aureus* strain Mu50. (A) The open reading frames flanking *fmtA* operon are highlighted, and the strategy of division of the promoter region is also highlighted. The numbering of the upstream and downstream elements on the *fmtA* promoter is based on the putative transcription starting point denoted by an asterisk and indicated in bold in panel B. The putative −10 box is also in bold.

The predicted promoter region of *fmtA* (P*fmtA*) lying between the open reading frames (orf) denoted SAV1056 and *fmtA*, encompassing 540 bp, was divided into three DNA fragments of 270 bp, each designated *seq1*, *seq2,* or *seq3* (Figure 1). The *seq1*, *seq2,* and *seq3* fragments were amplified from the *S. aureus* Mu50 genome (Cedarlane) using primers as follows (see Table 1): *seq1* (−342 to −70; the numbering of the upstream and downstream elements in the *fmtA* promoter is based on a putative transcription starting point (Figure 1B)) was amplified using Dir_seq1_ and Rev_seq1_, *seq2* (−70 to +199) with Dir_seq2_ and Rev_seq2_, and s*eq3* (−207 to +63) with Dir_seq3_ and Rev_seq3_. The PCR amplified fragments were gel-purified, 5′-end labeled with [γ-^32^P]ATP (3000 Ci/mmol) using T4 polynucleotide kinase, and purified by passage through ProbeQuant G-50 columns (GE Healthcare). Binding reactions were performed by mixing oxacillin-induced or uninduced cell extracts with 2 ng 5′-[^32^P]-labeled double-stranded DNA fragments in the presence of 1 µg poly (dI-dC) and 200 ng sheared herring sperm DNA for 30 min in 10 mM Tris-HCl (pH 7.5), 50 mM KCl, 1 mM dithiothreitol (DTT), 5 mM MgCl_2_, and 2.5% (v/v) glycerol. The reaction mixtures were loaded into 6% native polyacrylamide gels. Following electrophoresis, gels were dried and imaged using an electronic radiography instant imager (Packard). Quantitative analysis of the bands was performed using ImageJ (v 1.3, NIH). Dissociation constants were determined as the protein concentration that resulted in 50% bound DNA. The results were obtained from three independent binding experiments. To ascertain which region of *fmtA* promoter is important for binding to the SarA transcription factor, two additional DNA fragments were generated from the *fmtA* promoter region. These sequences, referred here in as *seqA* (−207 to −70) and *seqB* (−70 to +63), were amplified by PCR using specific primers pairs seqADir and seqARev for *seqA*, and seqBDir and seqBRev for *seqB* (see Table 1). Target DNA (2 ng) was mixed with purified SarA at concentrations varying from 1 nM to 1.8 µM in binding reactions for EMSA as described above for the cell extracts.

**Table 1:** The primers used in this study.

Primer Name	Primer Sequence
Dir_seq1_	5′ GAGAACCAATGCTAG AAGGATCAA 3′
Rev_seq1_	5′GACATCATACTAAAGTCGCATTAT 3′
Dir_seq2_	5′ TTAAAAAATTTAAAGGCGTAATAT 3′
Rev_seq2_	5′ TACACACG CATGTATAACTAGTTTT 3′
Dir_seq3_	5′ACTAATACATTATAGCCTTAAATTG 3
Rev_seq3_	5′ TT ATCATTCATTTATGCTTTACGCTA
Dir_seqB_	5′ ACTAATACATTATAGCCTTAAATTG 3′
Rev_seqB_	5′ GACATCATAC TAAAGTCGCATTAT 3′
Dir _seqC_	5′ TTAAAAAATTTAAAGGCGTAATAT 3′
Rev_seqC_	5′ TTATCAT TCATTTATGCTTTACGCTA 3′
SarADir	5′ CATATGGCAAT TACAAAAATCAATGATTGCTTTGAG 3′
SarARev	5′ AGCAAGCTTTTA TTATAGTTCAATTTCGTTGTTTG 3′
SarAMDir	5′ CGTAATGAGCATGA TGATGAAGCAACTGTATTAATTCTTG 3`
SarAMRev	5′ CAAGAATTAAT ACAGTTGCTTCATCATGCTCATTACG 3′
DirP_seq1_	5′ A*GGAATTC*G AGAACCAATGCTAGAAGGATCAA 3′
RevP_seq1_	5′ CG*GGATCC*GA CATCATACTAAAGTCGCATTAT 3′
DirP_seq2_	5′ AG*GAATTC*TTAAAAAATTTAAAGG CGTAATAT 3′
RevP_seq2_	5′ CG*GGATCC*TACACACGCATGTATAACTAGTTTT 3′
DirP_seq3_	5′ AG*GAATT*CACTAATACATTATAGCCTTAAATTG 3′
RevP_seq3_	5′ CG*GGATCC*TTATCATTCATTTATGCTTTACGCTA 3′
*luxA*	5′ TAAGCAAAAGTTTCCAAATTTCAT 3′
FmtAT1GDir	5′ AGGTATAATTAATTTTG*G*TGTATAGATAACAT 3′
FmtAT1GRev	5′ ATGTTATCTATACA*C*CAAAATTAATTATACCT 3′
FmtAT14GDir	5′ TACAAAGT*G*ATAAGGGATAATTAATTTTGTTG 3′
FmtAT14G Rev	5′ CAACAAAATTAATTATCCCTTAT*C*ACTTTGTA 3′
16S-RNADir	5′ GCTAAGTGTTAGGGGGTTTCC 3′
16S-RNARev	5′ TTCAACCTTGCGGTCGTACT 3′
SarADir	5′ TGTTTGCTTCAGTGATTCGTTT 3′
SarARev	5′ TCTTGTTAATGCACAACAACGTAA 3′
FmtADir	5′ TGGTACGAAAAAGTATCCAGATG 3′
FmtARev	5′ CCAAAGAATCCCCCGTTAAG 3′

### Screening Protocol for Isolation of Regulatory Proteins

The P*fmtA* derived sequence *seq3* (−207 to +63) was amplified by using 5′ biotinylated primers and their respective reverse primers (Table 1). An aliquot of 200 µg streptavidin-coated magnetic beads (10 µg/µL) (Dynal® Biotech) was washed three times with 20 µL washing buffer (10 mM Tris-HCl buffer pH 7.5, supplemented with 1 mM EDTA and 2 M NaCl) for 15 min at room temperature. A 40-µL aliquot of the *seq3* target DNA at 25 ng/µL was incubated with the beads for 15 min. The supernatant was removed and the beads were washed twice with the washing buffer. The process of loading target DNA on the beads was repeated two more times, with a washing step after each loading step. This process ensured 90% loading of the beads with the target DNA. Prior to incubation with DNA-bound beads, oxacillin-induced cell extracts were incubated with 200 µg streptavidin-coated beads in binding buffer (10 mM Tris buffer, pH 7.5, supplemented with 150 mM KCl, 0.1 mM EDTA, and 0.1 mM DTT) on ice for 30 min. The pre-treated cell extracts (40 µL) were then incubated with the DNA-loaded beads for 30 min at room temperature in the presence of 100 ng/µL herring sperm DNA.

Unbound protein was removed from the beads by three washes with 100 µL binding buffer. The beads were then resuspended in 25 µL water and subjected to trypsin digestion (EMD4 Biosciences). The samples were analyzed by liquid chromatography-tandem mass spectrometry (LC-MS-MS) at the Advanced Protein Technology Centre of Hospital for Sick Children (Toronto, Canada). Alternatively, the beads were resuspended in 25 µL water, mixed with 0.5 µL 10% sodium dodecyl sulfate (SDS) and boiled for 5 min. The supernatant was loaded into SDS-polyacrylamide gels.

### Cloning of *sarA*

The 375 bp *sarA* coding region was amplified from *S. aureus* strain MRSA252 by PCR using primers SarADir and SarARev (Table 1) which incorporate *Nde*I and *Hind*III restriction enzyme sites at the 5′-end and 3′-end respectively. The amplicon was digested with *Nde*I and *Hind*III and cloned into vector pET22b digested with *Msc*I and *Hind*III. The resulting plasmid was digested with *Nde*I (to remove the *pelB* leader sequence between the *Nde*I site of pET22b and the *sarA* gene), re-ligated, and transformed into *E. coli* NovaBlue cells. The pET22b::*sarA* construct was confirmed by sequence analysis, and transformed into *E. coli* BL21(DE3) cells.

### Site-directed Mutagenesis of Arg90 to Ala in SarA

The Arg90 residue of SarA was mutated to Ala using the QuickChange™ Site-Directed Mutagenesis protocol (Agilent, Mississauga, Canada). The recombinant DNA construct pET22b::*sarA*R90A was generated using pET22b::*sarA* as template, *Pfu* Turbo™ DNA polymerase (Agilent) and two mutagenic primers: SarAMDir and SarAMRev (Table 1). The PCR product was treated with restriction endonuclease *Dpn*I and the resulting mixture was used to transform *E. coli* NovaBlue cells. Successful mutation of arginine to alanine was verified by DNA sequencing and the pET22b::*sarA*R90A plasmid was used to transform *E. coli* BL21(DE3) cells.

### Purification of SarA and SarAR90A Mutant Proteins

All steps of purification were carried out at 4°C. Seed cultures in BL21(DE3) cells were grown overnight in Luria-Bertani media (Thermo-Fisher). A 1% starter culture was used to inoculate 1 L Terrific Broth (TB), supplemented with 100 µg/mL ampicillin. The culture was grown at 37°C with shaking until the OD_600 nm_ reached 0.7. SarA expression was initiated by adding 1 mM isopropyl-1-thio-β-D-galactopyranoside (IPTG; Rose Scientific, Edmonton, Canada). The induced cells were harvested and resuspended in 50 mM sodium phosphate buffer (pH 6.8). The protein was liberated by sonication and cellular debris removed by centrifugation at 20,000×*g* for 60 min. The clarified supernatant was loaded onto a SP-Sepharose FF column and the protein was eluted with a linear gradient of 1.0 M NaCl in 50 mM sodium phosphate buffer (pH 6.8). The fractions containing protein were concentrated and the buffer was changed to 20 mM Tris-HCl, pH 7.4, 1 mM EDTA, and 1 mM DTT using a HiPrep desalting column (26/10, GE Healthcare). The protein mixture solution was then loaded to a Heparin Sepharose 6FF column (26/10, GE Healthcare). The protein was eluted with a linear gradient of 1.5 M NaCl. The fractions containing protein were concentrated and loaded onto a Superdex^TM^75 (10/300 GL) column (GE Healthcare) equilibrated with 50 mM sodium phosphate buffer (pH7.0). Following elution by the same buffer, the protein-containing fractions were analyzed by 15% SDS-polyacrylamide gel electrophoresis (PAGE). The molecular mass of the purified SarA and SarAR90A mutant were confirmed by electrospray ionization mass spectrometry in the Advanced Protein Technology Centre of Hospital for Sick Children (Toronto, Canada).

The oligomerization state of SarA was determined by size exclusion chromatrography using a gel filtration LMW calibration kit from GE Healthcare and Superdex^TM^75 (10/300) column (GE Healthcare). The column was equilibrated and run with 50 mM sodium phosphate buffer (pH 7.0) at a flow rate of 0.4 mL/min.

### DNase I Footprinting Assay

The *seqA* and *seqB* promoter sequences were amplified by PCR from the *S. aureus* Mu50 genome using specific primers (Table 1). The primer of interest (Dir or Rev) was 5′–end labeled with [γ-^32^P] ATP (3000 Ci/mmol). Binding reactions were performed as described above for the EMSA, but with 8 ng DNA. Binding reactions were incubated for 30 min at 37°C and then subjected to DNaseI (0.024 units) for 2 min at 25°C after supplementing binding buffer with 10 mM CaCl_2_ and 5 mM MgCl_2_. The reaction was terminated by adding stop solution (200 mM NaCl, 1% (w/v) SDS, 20 mM EDTA, and 250 µg/mL tRNA). The digested DNA samples were extracted with phenol:chloroform and ethanol precipitated. They were then resuspended in formamide containing loading dye, denatured at 95°C and loaded onto 8% sequencing gel. The control A, T, C, and G were prepared by using Therminator DNA polymerase (New England Biolabs Canada) and a set of acyclonuleotides (acyATP, acyCTP, acyGTP, and acyTTP; New England Biolabs Canada). Four separate reactions (one for each acyNTP terminator) generated DNA sequencing termination ladders. Reactions contained template DNA, 50 nM [γ-^32^P] ATP labeled sequencing primer, 50 nM dNTP, 20 mM Tris–HCl, pH 8.8 at 25°C, 10 mM KCl, 2 mM MgSO_4_, 10 mM (NH_4_)_2_SO_4_, 0.1% Triton X-100, and 0.1 U/µL of *E. coli* RNA polymerase haloenzyme (Epicentre Biotechnologies, Madison, Wisconsin).

### *In vitro* Run-off Transcription Assay

Single round transcription by *E. coli* RNA polymerase holoenzyme (RNAP; Epicentre Biotechnologies, Madision, Wisconsin) was carried out using a 580-bp fragment of the *fmtA* promoter region (P*_fmtA_*_,_ nucleotide position −342 to +239 of *fmtA* promoter region) as template, in the absence or presence of SarA. The P*_fmtA_* fragment was amplified using *pfu* Turbo DNA polymerase and primers Dir-P*_fmtA_*, (5′-GAGAACCAATGCTAGAAGGATCAA-3′) and Rev-P*_fmtA_* (5′-TCGATGAAAAATTAACGCTATAGAAA-3′). The *in vitro* transcription reactions were performed as described previously with some modifications [38]. The DNA template (5 nM) was incubated with SarA (0.1 and 0.6 µM) in transcription buffer (40 mM Tris, pH 7.5, 150 mM KCl, 10 mM MgCl_2_, 2 mM DTT, 100 µg/mL BSA, and 0.05% Triton X-100) for 20 min at 30°C. One unit of *E. coli* RNAP (0.5 µg) was added to each reaction mixture and the reactions were incubated for another 20 min at 30°C. A control reaction containing only RNAP was prepared similarly. Transcription was initiated by adding 3 µL NTP mix containing 1 mM ATP, 1 mM CTP, 1 mM GTP, 100 µM UTP, 2 µCi of [α- ^32^P]-UTP, 600 µg/µL heparin, and 40 U/µL Murine RNase inhibitor. The reactions were placed at 30°C for 20 min. The reactions were terminated by addition of 5 µL transcription stop buffer (20 mM EDTA, 1% SDS, 1 mg/ml bromophenol blue, 1 mg/ml xylene cyanol, and 90% formamide). The samples were heated at 90°C for 5 min and immediately loaded onto an 8% polyacrylamide gel containing 7 M urea. The φX174 DNA/*HinfI* dephosphorylated DNA fragments (Promega), labeled with [γ-^32^P] ATP (3000 Ci/mmol), were used as a molecular weight marker. The gels were dried and exposed on phosphor screen and visualized using a Typhoon Trio^+^ Variable Mode Imager (GE Healthcare).

A transcription start point in *fmtA* promoter was identified the T residue located at the 156^th^ nucleotides upstream of the *fmtA* start codon (Figure 1). A −10 box sequence of 5′-TATAAT-3′ sequence is present relative to that start site, which is identical to the −10 sequence recognized by the *E. coli* σ^70^ RNAP [39]. To investigate the role of the proposed transcription initiation elements, we designed two mutations in the *fmtA* promoter sequence and assessed their role in transcription by transcription run-offs experiments. In the mutant P*_fmtA_*_T1G_, we replaced the proposed transcription start site T (+1) with a G residue. In the P*_fmtA_*_T14G_ mutant promoter, we substituted the T at −14 with a G residue because this T/A pair at the beginning of the - 10 consensus sequence is critical in interactions with RNAP [40]–[42]. To accomplish the mutagenesis, we cloned P*_fmtA_* sequence into the pSTBlue vector (Invivogen). The mutagenic primers are presented in Table 1.

### Construction of *luxABCDE* Fusion Strains with Derivatives of *fmtA* Promoter Region

The three P*fmtA-*derived sequences (*seq1, seq2* and *seq3*) were amplified by PCR from *fmtA* operon using specific primer pairs designed to introduce *Eco*RI and *Bam*HI restriction sites at the 5′ and 3′ ends, respectively (Table 1). The predicted full length *fmtA* control region encompassing nucleotides −342 to +199 (referred to as *seq1–2*) was also amplified using primers DirP_seq1_ and RevP_seq2_. All four DNA fragments, P_seq1,_ P_seq2,_ P_seq3,_ and P_seq1–2_, were PCR amplified and digested using restriction enzymes *Eco*RI and *Bam*HI. The DNA fragments were ligated to similarly digested vector pXEN1. pXEN1 harbors the *luxABCDE* operon from *Photorhabdus luminescens* that has been modified for *lux* expression in Gram positive bacteria [7]. The ligation mix was transformed into *E*. *coli* DH5α and transformants were selected on medium containing ampicillin (100 µg/mL). Putative clones obtained were confirmed to harbor the inserts by sequence analysis using a primer derived from *luxA* and the forward primer used to amplify the insert fragment. The pXEN1 plasmids carrying different inserts are referred to as *lux* fusion plasmids.

The pXEN1 plasmid and *lux* fusion plasmids were introduced into the restriction-deficient *S. aureus* strain RN4220 by electroporation (26). Transformants were plated on tryptic soy broth (TSB)-agar plates supplemented with 5 µg/mL chloramphenicol (selectable marker in pXEN1). Positive colonies were confirmed by sequencing of the plasmids using the primers described above. The *S. aureus* strain RN4220 carrying pXEN1 is referred to as RN(::*lux*), and RN4220 strains carrying the pXEN1 fusion plasmids, constructed herein, are referred to as *lux* fusion strains RN(P_seq1_::*lux*), RN (P_seq2_::*lux*), RN(P_seq3_::*lux*), and RN(P_seq1–2_::*lux*).

In *S. aureus* PC1839 (parent strain *S. aureus* RN4220), the *sarA* gene has been knocked out and replaced with a kanamycin resistance cassette [43]. PC1839 was transformed with the *lux* promoter constructs P_seq1,_ P_seq2,_ P_seq3,_ P_seq1–2_, and pXEN1 isolated from strain RN4220. The resultant colonies were resistant to kanamycin and chloramphenicol. The *S. aureus* strain PC1839 carrying the empty vector pXEN1 is referred to as PC(::*lux*), and PC1839 strains carrying the pXEN1 fusion plasmids are referred to as *lux* fusion strains PC(P_seq1_::*lux*), PC(P_seq2_::*lux*), PC(P_seq3_::*lux*), and PC (P_seq1–2_::*lux*). The effect of pXEN1 and *lux* fusion plasmids on the growth of *S. aureus* RN4220 and PC1839 fusion strains was investigated by monitoring the growth profiles. Briefly, *lux* fusion strains RN(::*lux*), PC(*::lux*), RN4220, and PC1839 were inoculated into TSB and grown overnight at 37°C with 5 µg/mL chloramphenicol, except for RN4220 and PC1839. Next, 1% culture was inoculated in fresh TSB supplemented with 5 µg/mL chloramphenicol for the *lux* fusion strains except for RN4220 and PC1839. Then the OD(_600 nm_) was measured at 1 h intervals for the next 7 h.

### Measuring Bioluminescence from *S. aureus* Strains

After overnight growth, RN4220, PC1839, RN(::*lux*), and PC(::*lux*), were diluted into fresh TSB and grown at 37°C with shaking at 200 rpm. The strains were grown to an OD_600 nm_ of approximately 0.3 and induced with oxacillin at 10 µg/mL or 100 µg/mL for 1 h. The optical densities were measured at 600 nm for all the samples. The cultures with higher OD(_600 nm_) were diluted such that the cell density in each culture was the same. A 300-µL aliquot from each sample (in triplicates) was transferred to opaque 96-well optiplates and analyzed in a HT-Analyst (Molecular Devices, California). Bioluminescence was measured immediately after dispensing the samples into the plates over a period of 10 min. The data points collected over 10 min were averaged for each strain at each oxacillin concentration, and the standard deviations were determined from three independent measurements. The promoter activity was plotted as luminescence over time using the mean values from the readings taken over a period of 10 min calculated by subtracting values from blank medium controls. These experiments were repeated three times.

### Viability Testing of the Induced Versus Uninduced Cultures

Cultures of RN4220, PC1839, RN(::*lux*), and PC(::*lux*) were inoculated at 1∶100 with an overnight culture into TSB with chloramphenicol (no antibiotic was added to RN4220 and PC1839). When the OD_600 nm_ of the cultures reached approximately 0.3, oxacillin was added to a final concentration of 10 or 100 µg/mL, and the cultures were grown for 1 more hour. The OD of each culture was normalized to the sample having the lowest OD. A 20-µL aliquot from each culture was diluted 5000-fold with TSB to dilute the antibiotic, and a 50-µL aliquot of the dilution was plated on TSB-agar plates without antibiotic and incubated at 37°C. These experiments were repeated three times.

### RNA Extraction and c-DNA Synthesis

Overnight-grown wild-type *S. aureus* RN4220 and PC1839 were diluted (200-fold) separately in 30 mL tryptic soy broth (TSB) in 125-mL Erlenmeyer flask. Cultures were incubated at 37°C with shaking at 200 rpm and growth was measured at regular intervals until the optical density (OD_600_ nm) reached to early-exponential phase (∼0.4). Cultures were then distributed in 2 flasks (10 mL each), where one culture flask was used as control, and other was treated with 10 µg/mL of oxacillin for 15- and 60-min. Control cultures were incubated along with the treated culture for 15-min and 60-min. Aliquots of 2 mL cultures from each flask were taken, and mixed with 4 mL of bacterial RNA Protect solution (Qiagen, Valencia, CA). The mixtures were further vortex briefly for 5 s and incubated for 5 min at room temperature, followed with centrifugation at 5000×*g* for 20 min in a swinging-bucket rotor centrifuge to collect the cells. Cells were washed with fresh TSB and used for isolation of RNA.

Cells were further suspended in 200 µL of TE (30 mM Tris-HCl; 1 mM EDTA, pH 8.0) buffer containing lysostaphin (50 µg/mL), mixed by vortexing for 10 s, and incubated at room temperature for 10 min on a shaker–incubator. Subsequently, 10 µl Proteinase K (100 µg/mL, solution) was added in the solution and total RNA was extracted and purified using an RNeasy minikit (Qiagen) following the manufacturer recommendation for bacterial RNA isolation. For RNA isolation of both antibiotic treated and untreated cultures, two independent bacterial cultures were prepared. cDNA was synthesized using a high capacity RNA-to-cDNA kit (Applied Biosystems, Foster City, CA). RNA concentrations were determined by absorbance readings at 260 and 280 nm, using a Nanodrop ND-1000 UV spectrophotometer (Nanodrop Technologies, Wilmington, DE).

### Quantitative Real-time PCR (qRT-PCR)

qRT-PCR was used to characterize the transcript levels in the cells in response to oxacillin treatment. qRT-PCR was performed with Rotor-Gene Real-Time PCR Cyclers (Qiagen) using SYBR green technology. RT-PCRs were performed in a 20 µL reaction volume containing 1 µL of template DNA (25 ng), 1 µL of gene specific primers (10 µM, Table1), 10.0 µL of Power SYBR green PCR Master Mix (Applied Biosystems, Foster City, CA) and 8.0 µL of H_2_O. The following PCR conditions were used: 50°C for 2 min, 95°C for 10 min, 40 cycle of 95°C for 15 s, 60°C for 30 s, 72°C for 30 s and one final extension step of 72°C for 10 min. The transcript levels of the *fmtA* and *sarA* genes were normalized using the *16S rRNA* transcript level of *S. aureus* RN4220 as an internal control. Each gene assay was performed in triplicate. The data analysis was carried out with Rotor-Gene Q software (Qiagen).

### Determination of MIC

Minimum inhibitory concentration (MIC) assays were performed as follows. Oxacillin dilutions were made using sterilized water and then aliquotted (1.5 µL) into 96-well plates. An overnight culture of RN4220 and *sarA* mutant were diluted 1/1000 into fresh TSB medium to give ca. 5×10^5^ CFU mL^−1^, and 150 µL aliquots were added to each well of the 96-well plate. Plates were incubated at 37°C for ∼18 h. MIC determinations were carried out on two different times in duplicate.

## Results

### Screening for the Transcription Factor(s) of *fmtA*

**Figure 2:**
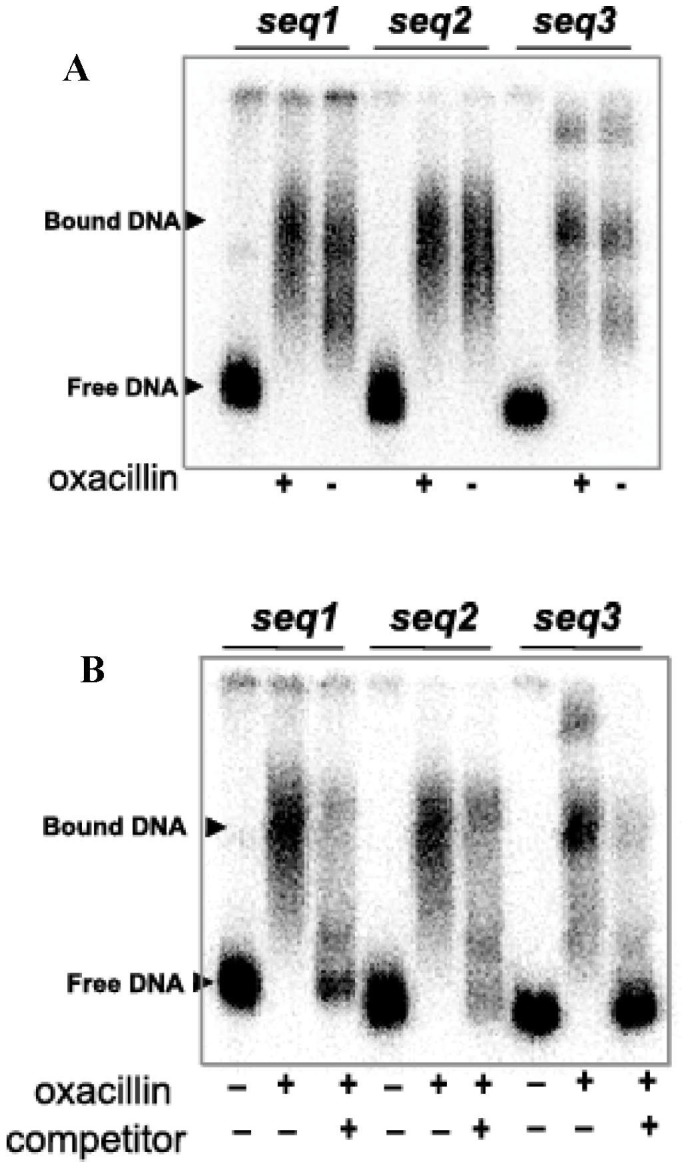
Probing for *fmtA* transcription factor(s) in *S. aureus* cell extracts. (A) Binding reactions between 5′-^32^P-end labeled *seq1, seq2*, and *seq3* fragments of P*fmtA* and oxacillin-induced (+) or uninduced (-) *S. aureus* cell extracts. (B) EMSA of binding reactions between 5′-end [γ-^32^P] ATP labeled *seq1*, *seq2* and *seq3* (2 ng) and oxacillin-induced *S. aureus* cell extracts in the presence and absence of unlabeled competitor (400 ng).

We divided the predicted 540 bp *fmtA* promoter region into three DNA fragments of approximately 270 bp each, referred to as *seq1*, *seq2,* and *seq3*, with *seq3* overlapping *seq1* and *seq2* at the 5′ and 3′ ends respectively (Figure 1). When radioactively labeled *seq1*, *seq2,* and *seq3* were incubated with oxacillin-induced and uninduced cell extracts from *S. aureus* RN4220, electrophoretic mobility shifts were observed (Figure 2A). Presence of oxacillin has a clear effect on the electrophoretic mobility profile of DNA considering the multitude of non-specific interactions that these DNA fragments could be subject of. Further, introduction of unlabeled DNA targets eliminated the mobility shifts of the labeled target DNA fragments (Figure 2B). These observations suggest that the promoter region of *fmtA* is the target of regulation by transcription factors.

We developed a screening protocol to isolate the transcription factor(s) of *fmtA*, using biotinylated *seq3* (−207 to +63) immobilized on streptavidin-coated magnetic beads. These beads when incubated with oxacillin-induced cell extracts pulled down several proteins that were identified by analyzing LC-MS-MS data using the MASCOT program and a probability based Mowse score of >45. The analyses showed the presence of staphylococcal accessory regulator A (SarA), pyruvate carboxylase, biotin carboxyl carrier protein of acetyl-CoA carboxylase, DNA-direct RNA polymerase alpha chain, DNA polymerase I, translation elongation factor Tu, translation elongation factor G, elongation factor TS, chaperone protein dnaK, chaperonin GroEL, cell division proteins FtsZ, DNA binding protein II, putative DNA-binding protein, and 30S ribosomal protein S5.

To confirm that SarA is pulled down by this protocol we incubated the oxacillin-induced cell extracts with *seq3* and boiled the magnetic beads in SDS-loading buffer after they were washed twice with washing buffer. Then, we loaded the supernatant to a 15% SDS-PAGE. The SDS-PAGE was stained by commassie blue, and the gel in the region of 14 kD was cut and subjected to trypsin digestion and LC-MS-MS. This process resulted in only one hit, SarA. Seven SarA peptides were identified, which covered 69% of the protein sequence (79/113 amino acids). We then focused on the SarA protein, a 14-kD global regulatory protein involved in regulation of virulence factors [44], to investigate the transcriptional regulation mechanism of *fmtA*.

### Purification of SarA and Characterization of its Oligomerization State

**Figure 3:**
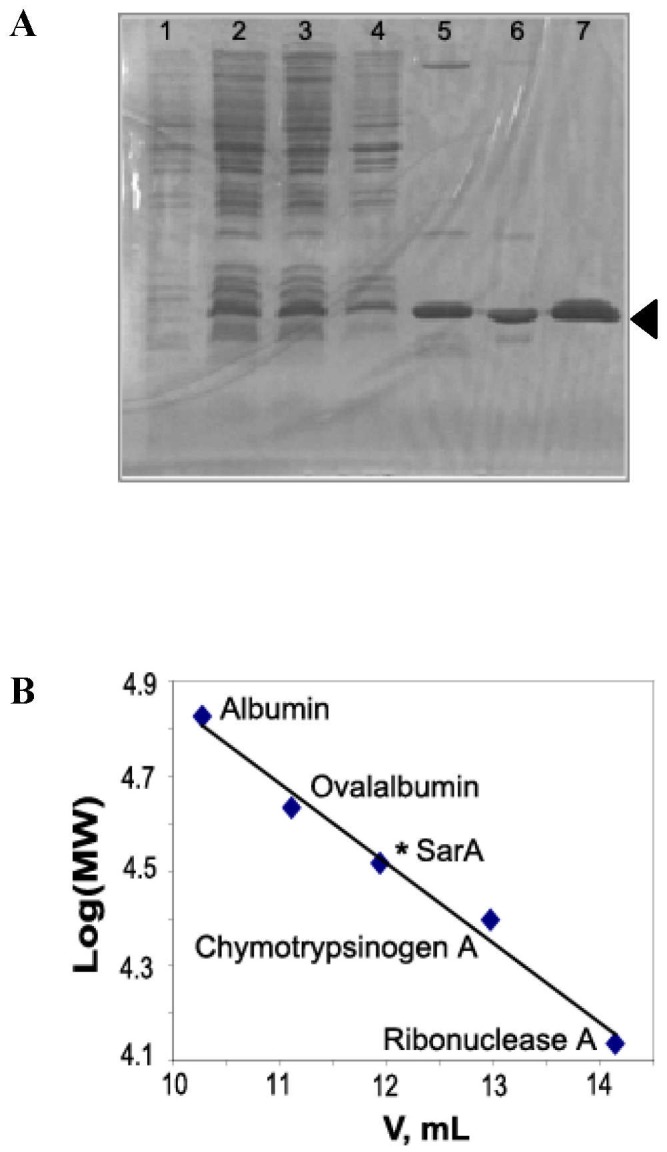
Analysis of SarA purification and oligomerization. (A) Coomassie Blue stained 15% SDS-PAGE indicating SarA at each step of purification (the black arrow). Lane 1, whole cell extract before induction; Lane 2, whole cell extract after induction by IPTG; Lane 3, soluble fraction of cell lysate after sonication; Lane 4, insoluble fraction of cell lysate after sonication; Lane 5, SarA after SP Sepharose column; Lane 6, SarA after Heparin Sepharose column; Lane 7, SarA after size exclusion column. (B) Four proteins: Ribonuclease A (13.7 kD), Chymotrypsinogen A (27.0 kD), Ovalalbumin (43.0 kD) and Albumin (67.0 kD) were used to calibrate a Superdex™ 75 (10/300 GL) column in order to determine the oligomerization state of SarA in solution. The medium of the column was 50 mM sodium phosphate (pH 7.0) and it was run at a flow rate of 0.4 mL/min.

SarA belongs to the family of transcription factors called the “wing-helix” proteins [45]. Full-length SarA gene was cloned, expressed, and purified to homogeneity (Figure 3). The identity of the protein was confirmed by mass spectrometry. The observed molecular mass of SarA was 14,635 D (theoretical mass, 14,586 D).

The oligomeric state of SarA was investigated using gel filtration chromatography. Based on the elution times of four standards, the molecular mass of SarA was estimated to be 30 kD (Figure 3B), which shows that SarA is dimer in our solution as shown previously [45], [46], hence we are working with a functional protein.

### SarA Binding to P*fmtA*

**Figure 4:**
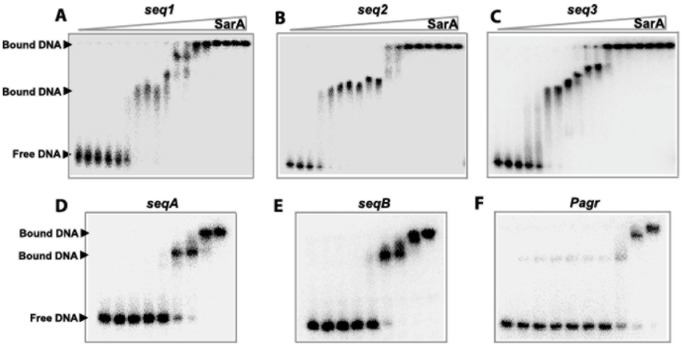
EMSA-based analysis of the DNA binding activities of SarA. 5′-[^32^P]-end labeled *seq1* (A), *seq2* (B), *seq3* (C) fragments of P*fmtA* were incubated with increasing amounts of SarA (15, 23, 30, 45, 60, 75, 90, 105, 120, 150, 225, 300, 450, 600, 900, 1200, 1500, and 1800 nM). In the bottom panels, 5′-[^32^P]-end labeled *seqA* (D) and *seqB* (E) fragments of P*fmtA* were incubated with increasing amounts of SarA (0, 15, 30, 60, 120, 240, 480, 960, and 1500 nM). The 5′-[^32^P]-end labeled *agr* promoter region (F) was incubated with 30, 60, 90, 120, 180, 240, 480, 960, and 1800 nM SarA.

To elucidate whether purified SarA binds directly to the P*fmtA* promoter, we performed EMSA experiments with *seq1*, *seq2*, *seq3*, *seqA* and *seqB* (Figure 1). The dissociation constant of each DNA sequence was determined as the concentration of SarA that resulted in 50% DNA bound. We estimated the dissociation constants of SarA with respect to *seq1*, *seq2*, *seq3*, *seqA*, and *seqB* to be 63±9 nM, 54±12 nM, 66±7 nM, 147±18 nM, and 139±17 nM, respectively (Figure 4). As a control, we used a 45-bp sequence derived from the promoter region of *agr* that harbors the SarA binding consensus sequence (underlined), P*agr*: 5′-GTAAATTTTTTTATGTTAAAATATTAAATACAAATTACATTTAAC-3′ (*47*). The SarA binding affinity for the P*agr* sequence was about 400 nM (Figure 4).

Supershifts were observed in the EMSA experiments with *seq1*, *seq2*, *seq3*, *seqA*, *seqB,* and P*agr* at SarA concentrations higher than 300 nM (Figure 4). Interestingly, in the case of *seq3*, which covers the 3′-end of *seq1* and 5′-end of *seq2*, the supershift starts at 150 nM SarA. This observation indicates that either there is more than one SarA binding site in these sequences, which are saturated at higher protein concentrations, or there are different oligomers of SarA binding to the fragments; SarA is known to form dimers and dimers of dimers in target promoters and is proposed to oligomerize at its binding sites (*45*).

### Identification of SarA Binding Sites on P*fmtA* by DNase I Footprinting

**Figure 5:**
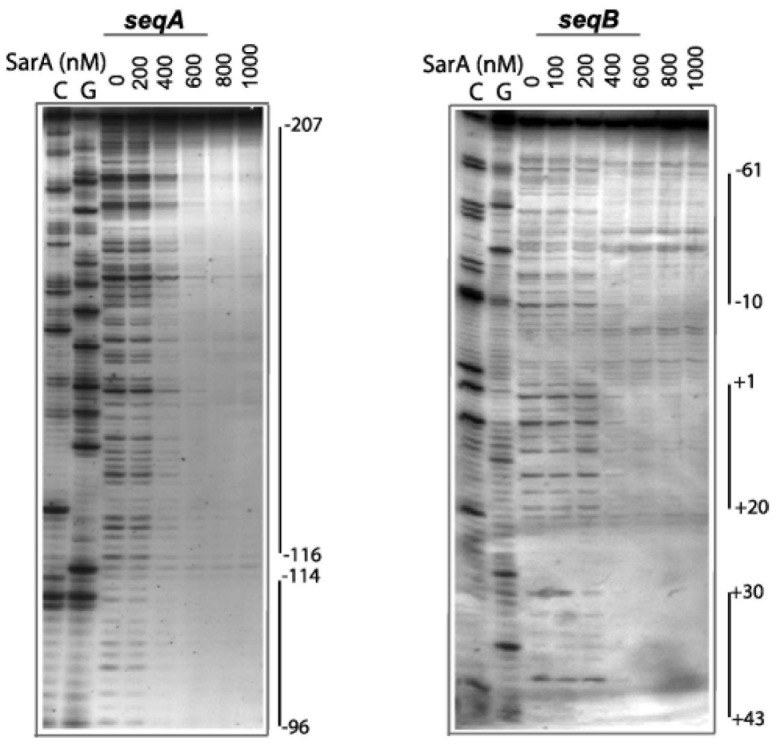
DNaseI footprint analysis of SarA binding to *seqA* and *seqB* fragments of P*fmtA*. A. Each DNA sequence was labeled with [γ-^32^P]ATP on the 5′-end of the bottom strand.

**Figure 6:**
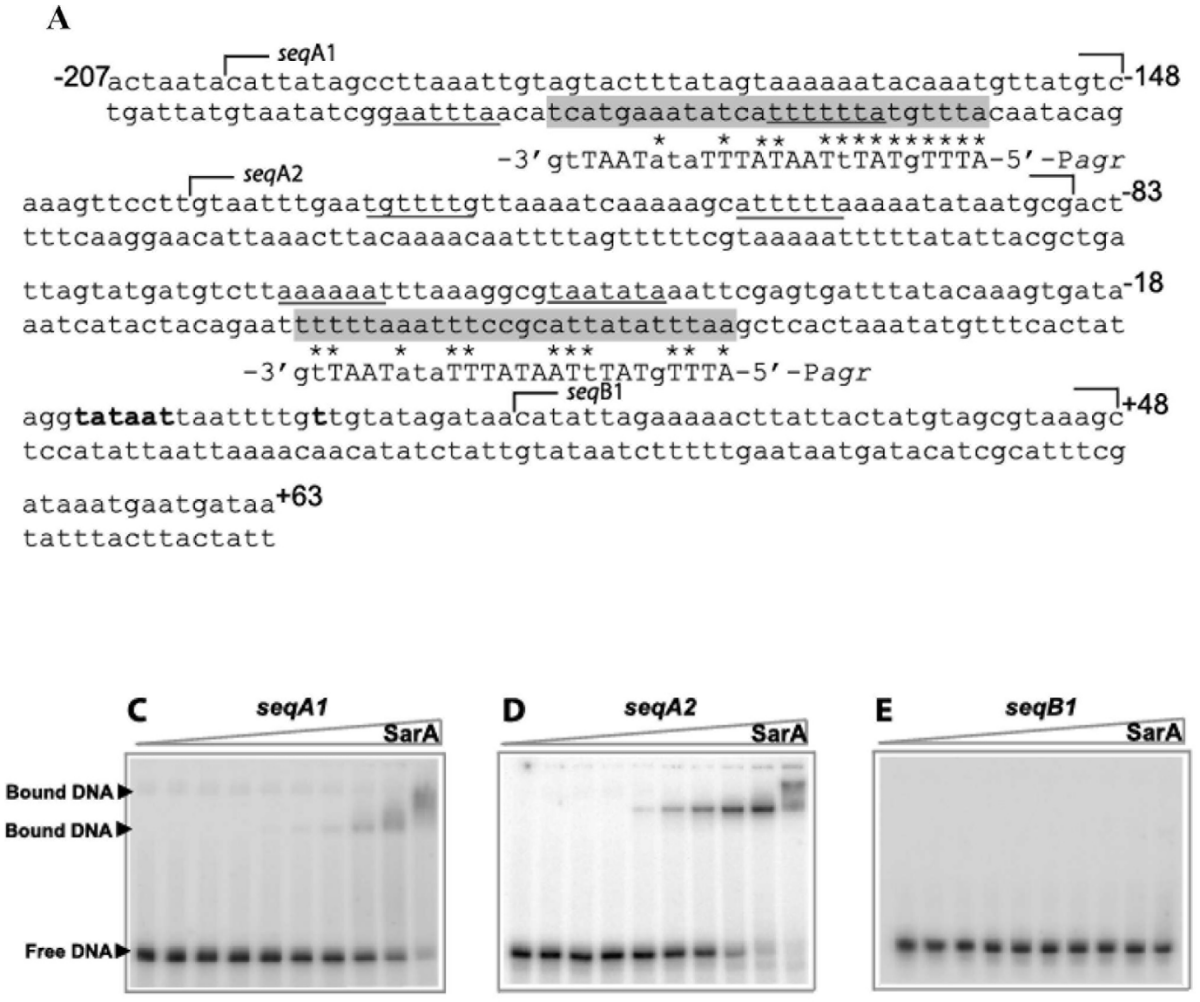
SarA binding specificity to P*fmtA*. (A) The DNA sequence of the P*fmtA*-*seq3* region. The 26-bp SarA DNA-binding consensus sequence identified in this region of P*fmtA* is highlighted in gray. The highlighted areas are aligned with the 26-bp SarA binding sequence of the *agr* promoter. The nucleotides proposed to be the conserved SarA binding sites are in capital letters. The asterisks indicate the nucleotides shared between the SarA consensus binding sequence and *fmtA*. The 7-bp binding sequence for the winged-helix-turn-helix proteins are underlined. The various *fmtA* promoter fragments (seqA1, seqA2 and seqB1) used in the EMSA studies are also indicated. (B) The EMSA of the 5′-[^32^P]-end labeled *seqA1 fragment* incubated with 0, 60, 120, 180, 240, 360, 480, 960, 1800 and 3600 nM SarA. (C) The EMSA of the 5′-[^32^P]-end labeled *seqA2 fragment* incubated with 0, 60, 120, 180, 240, 360, 480, 960, 1800 and 3600 nM SarA. (D) The EMSA of the 5′-[^32^P]-end labeled *seqB1 fragment* incubated with 0, 30, 60, 90, 120, 180, 240, 480, 960, and 1800 nM SarA.

Two fragments of P*fmtA* spanning the regions −207 to −70 (*seqA*) and −70 to +63 (*seqB*) were used to investigate SarA binding to P*fmtA* (Figure 1). The SarA protection sites on the bottom strand of each sequence are shown in Figure 5. SarA provided a protection of the DNA at concentrations higher than 200 nM. A similar DNase I protection profile was observed on the top strand of each DNA sequence (data not shown). Alignment of these sequences against the proposed 26 bp SarA consensus binding site, 5′-ATTTGTATTTAATATTTATATAATTG-3′ [47], revealed that the SarA-protected sites in P*fmtA* encompass the SarA consensus binding site (Figure 6). Interestingly, the SarA-protected sites in P*fmtA* also included the 7-bp DNA sequence 5′-ATTTTAT-3′, recognized by the winged-helix proteins, and also suggested to be recognized by SarA [45], [48] (Figure 6A).

Additional three DNA fragments (obtained through primer annealing) were derived from the *fmtA* promoter region to investigate the putative SarA binding sites, referred to as *seqA1*, *seqA2* and *seqB1*, (Figure 6). The *seqA1* fragment harbors the 26-bp SarA consensus binding sequence identified in *seqA*, whereby the nucleotides −186 to −178 were omitted. The *seqA2* fragment harbors two 7-bp SarA binding sequences and *seqB1* is downstream of the putative transcription starting point (Figure 6A). The SarA binding affinities determined for *seqA1* fragment is ≥1.8 µM (no binding saturation was observed up to 3.6 µM) and 550 nM for *seqA2* fragment. No SarA binding was observed to *seqB1* at concentrations as high as 1.8 µM SarA (Figures 6B to 6D). The lower binding affinity for *seqA1* in comparison to P*agr* could be due to the removal of the four nucleotides however binding of SarA to *seqA1* suggests that the 26 bp SarA consensus binding site identified in *seqA1* recruits SarA and these nucleotides may be involved in the SarA binding.

### Analysis of the DNA-binding Properties of SarAR90A Mutant

**Figure 7:**
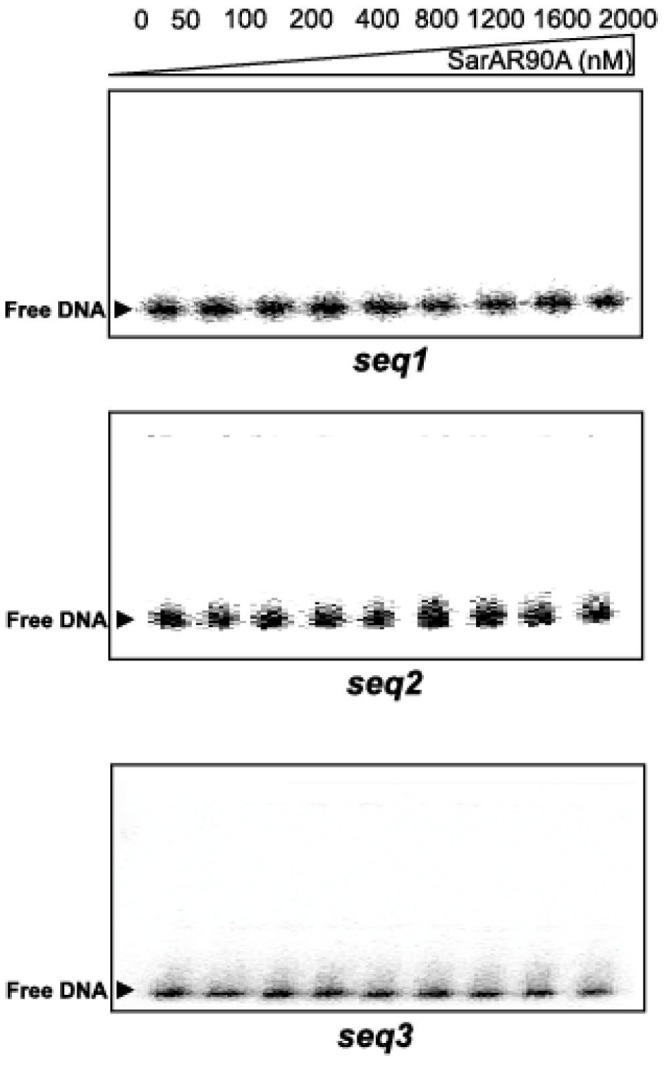
DNA binding activity of SarAR90A mutant. Binding reactions between [γ-^32^P]-end labeled *seq1*, *seq2* and *seq3* fragments of P*fmtA* and increasing amounts of SarAR90A variant.

Arginine at position 90 is essential for the SarA regulatory activity *in vivo*. It is located within the winged region of SarA and is part of the conserved basic region of SarA, the motif DER (*45*). We mutated this residue to alanine, purified the mutant protein, and confirmed the substitution by mass spectrometry. We then analyzed the binding affinity of the SarAR90A mutant protein to *seq1, seq2* and *seq3* by EMSA. The mutant protein failed to bind to these three DNA sequences (Figure 7).

### SarA Promotes *in vitro* Transcription of *fmtA*

**Figure 8:**
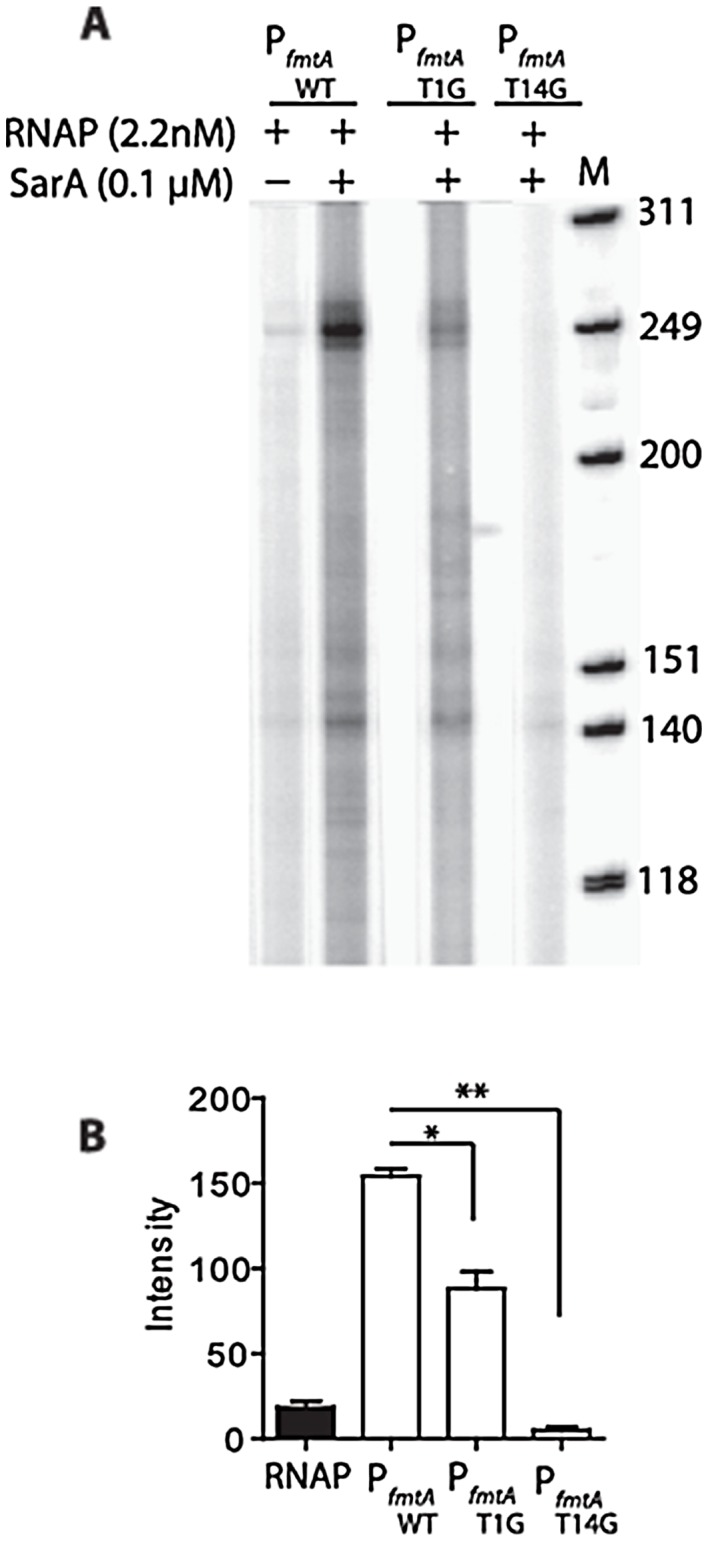
SarA activates *fmtA* transcription *in vitro*. (A) *In vitro* run-off transcription from the *fmtA* promoter region using *E. coli* RNAP holoenzyme and SarA. P*fmtA* region −342 to +199 was used as a template. The size of the transcript is compared against the φX174DNA/*Hinf* I molecular weight marker (M). (B) The intensities of the corresponding transcripts were measured by ImageJ software. The substitution of T1G in the proposed transcription start point of the *fmtA* promoter led to a significant decrease in the transcript (*p = 0.0242) whereas the mutation of the T14 to G in P_fmtA_T14G has the most pronounced effect on the transcription (**p = 0.001). The statistical analysis was carried out by student t test and GraphPad Prism software (La Jolla, California).

We used *E. coli* RNA polymerase holoenzyme (RNAP) in these experiments. Several lines of evidence lead us to conclude that *E. coli* RNAP is a good substitute for *S. aureus* RNAP in the case of *fmtA*. The *E. coli* RNAP is a complex of the RNAP core enzyme and the β^70^ factor protein. The DNA sequence of the putative −10 box in the *fmtA* promoter is identical to the consensus −10 box recognized by *E. coli* β^70^ (Figure 1), suggesting that the *fmtA* promoter may recruit a β^70^-like factor for transcription. The β^70^ homolog in *S. aureus* is SigA factor [49], [50], in turn suggesting that regulation of *fmtA* could be SigA-dependent. Incidentally, SarA regulates its own promoter in a SigA-dependent mechanism [51]. Our hypothesis that *E. coli* RNAP is a good substitute for that of *S. aureus* was confirmed by *in vitro* run-off transcription experiments with P*ftmA* and P*fmtA* mutated at the putative +1 and −14 sites. We observed that while *E. coli* RNAP could poorly initiate transcription from P*_fmtA_*, there was a complete lack of transcription for the T(+1) to G and T(−14) to G P*fmtA* variants (Figure 8). Furthermore, the presence of SarA increased the production of the transcript from the P*fmtA* fragment in a concentration-dependent manner (data not shown).

### SarA Regulates Expression of *fmtA–lux Fusion in vivo*

To investigate the role of the *fmtA* promoter in recruiting SarA *in vivo,* we fused P*fmtA*-derived sequences (P_seq1_, P_seq2_, P_seq3_, and P_seq1–2_) upstream of the *lux* operon in the reporter vector pXEN1 [52]. The *lux* fusion plasmids and the control vector pXEN1 were introduced into *S. aureus* RN4220. We hypothesized that SarA exerts direct regulation of *fmtA* via the SarA binding sites identified in our *in vitro* experiments. If this hypothesis were correct, we would expect that the disruption of SarA would reduce *lux* operon expression.

**Figure 9:**
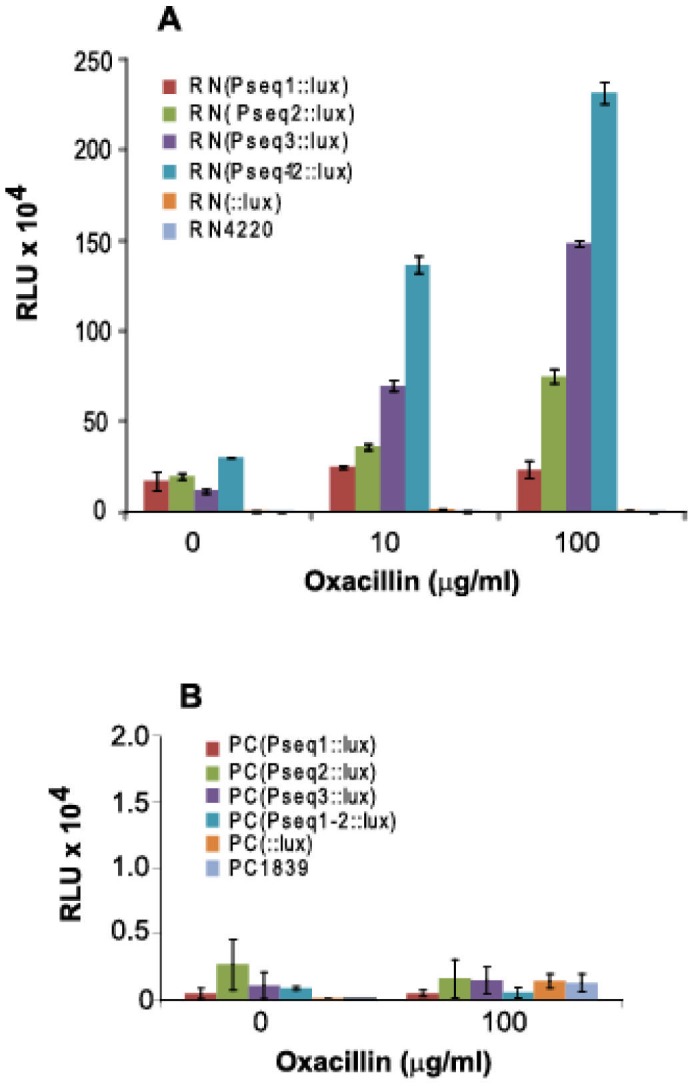
Probing the role of SarA on *fmtA* expression using a *lux* reporter vector. Luminescence signal recorded in the absence and presence of 10 or 100 µg/mL oxacillin. Each strain is represented by the name of the promoter fused upstream of the *lux* operon in the pXEN1 plasmid. A) Experiments carried out with *S. arueus* RN4220 strain and B) experiments carried out with *S. aureus* β*sarA* mutant strain, PC1839. The luminescence signals were recorded in triplicates and normalized based on the viability data. The error bars represent the standard deviations determined from these trials.

To test this hypothesis, we introduced the *lux* fusion plasmids into a SarA-deficient strain, PC1839 [53], and compared the luminescence of the RN4220 (*sarA* ^+^) strains and PC1839 (*sarA* ^-^) strains under oxacillin-induced and uninduced growth conditions. Strains harboring different fusion constructs showed comparable growth rates (data not shown). The luminescence data are shown in Figure 9. In the absence of oxacillin, a low level of luminescence signal over the background level, set as the RN4220::*lux*- strain, was observed in any lux-fusion strain. Presence of 10 µg/ml oxacillin increased the *lux* operon expression especially in the case of the *seq*3::*lux* and *seq*1–2::*lux* fusions, where a 5.8 and 4.5-fold increase in luminescence was observed, respectively, in comparison to the *lux* expression in the absence of oxacillin. The same trend was observed in the presence of 100 µg/ml oxacillin. Overall, luminescence of RN(P_seq1–2_::*lux*) and RN(P_seq3_::*lux*) exhibited the highest increase in luminescence in the presence of oxacillin. In contrast, none of the *sarA* mutant strains [PC(P_seq1_::*lux*), PC(P_seq2_::*lux*), PC(P_seq3_::*lux*) or PC (P_seq1–2_::*lux*)] showed any significant luminescence, even at 100 µg/ml oxacillin.

### Deletion of *sarA* Enhances *S. aureus* Sensitivity to Oxacillin

To understand whether the lack of functional SarA protein alters the sensitivity towards oxacillin, we tested the oxacillin MICs of wild type *S. aureus* RN4220 and *sarA* mutant. The *sarA* mutant showed modest, but significant decrease (2-fold) in MIC. The measured MICs of oxacillin for wild-type RN4220 and *sarA* mutant were 0.3 µg/mL and 0.15 µg/mL, respectively.

### Transcript Levels of *sarA* and *fmtA* in *S. aureus* RN4220 and PC1839

Our data revealed that *sarA* transcript level in *S. aureus* RN4220 did not change upon oxacillin treatment compared with corresponding untreated control culture. However, *fmtA* transcript level was increased 3-fold upon oxacillin treatment (3.5±0.7). This finding is in good agreement with the previously published data where no change in *sarA* transcript level, but increased transcript level of *fmtA* was observed [28]. Increased expression of *fmtA* in response to several cell wall active antimicrobials was observed in several studies [32], [54]. On the other hand, in the *sarA* mutant strain, PC1839, the transcript level of *fmtA* did not change upon oxacillin treatment compared with corresponding untreated control culture.

## Discussion

Genome-based studies have shown that *fmtA* levels in *S. aureus* are upregulated in the presence of cell wall inhibitors and by the knock-out of the genes involved in peptidoglycan biosynthesis [27], [28], [30], [55]. A study by Kuroda et al. suggested that *fmtA* is under the regulation of the two component signal transduction system VraSR [55]. However, the mechanism by which *fmtA* expression is regulated remains unknown. In our study DNA fragments derived from P*fmtA* region showed retardation in their electrophoretic mobilities when incubated with cell extracts isolated from *S. aureus* RN4220 with clear differences from the instance when bacteria were subjected to oxacillin (Figure 2A). The electrophoretic mobility shifts were eliminated in the presence of a competing DNA fragment (Figure 2B).

Herein, we report a screening protocol designed to identify the transcription factor(s) involved in regulation of *fmtA* expression. A 269-bp fragment of the *fmtA* promoter region (*seq3*) was immobilized on streptavidin-coated beads and used to capture proteins from extracts of *S. aureus* cells treated with oxacillin to induce *fmtA* expression. Protein binding and washing steps were performed at physiological ionic strength and in the presence of nonspecific DNA to reduce nonspecific interactions. A relatively small number of non-DNA-binding proteins were identified in the screen, including pyruvate carboxylase and biotin carboxyl carrier protein of acetyl-CoA carboxylase which both have biotin as a cofactor and could be binding to the beads through streptavidin. The presence of RNA polymerase alpha chain, DNA polymerase I, translation elongation factor Tu, translation elongation factor G, elongation factor TS, and 30S ribosomal protein S5 could be due to the presence of the *fmtA* ribosomal binding site in *seq3*. The presence of DNA binding protein II, a protein that recognizes polyA DNA regions, could bind nonspecifically to the *seq3* fragment which contains several polyA sites, and proteins such as chaperone protein DnaK, GroEL and FtsZ could bind nonspecifically to DNA or streptavidin.

SarA was the only transcription factor identified by our screening protocol. DNase I footprinting demonstrates that SarA binds to specific sites in the *fmtA* promoter region. Specificity of SarA binding to P*fmtA* was probed by mutation of one of the conserved residues located in the winged region of SarA, Arg-90. The mutant SarAR90A protein, which is unable to regulate the SarA target promoters *in vivo* [45], failed to bind to the *fmtA* promoter region (Figure 7).

The role of SarA in regulation of *fmtA* was corroborated by *in vivo* studies. Fusion of the *lux* operon to the various regions of the *fmtA* promoter indicated that the *fmtA* promoter region harbors transcriptional regulatory elements and that SarA is involved in the regulation of *fmtA* expression. These data are corroborated by the qRT-PCR data, whereby the *fmtA* expression level increases by 3.5-fold in the presence of oxacillin, while *sarA* level remains unchanged. In the *sarA* mutant, *fmtA* level are not affected by the presence of oxacillin. Interestingly, the *in vivo* data (Figure 9) suggest that SarA is involved in basal expression of *fmtA* as well as upregulation of *fmtA* in the presence of cell wall stress. Further, *in vitro* run-off studies suggest that transcription of *fmtA* may require the *S. aureus* primary sigma factor, SigA. This is in agreement with a previous report that shows that SarA regulates promoters that are SigA-dependent [51]. Additional evidence of the involvement of SigA in the transcriptional regulation of *fmtA* is provided by mutagenesis studies in which mutation of the −14 position and the transcription starting site of P*fmtA* resulted in drastic reduction of *fmtA* transcription.

Sequence alignment of P*fmtA*-*seq3* against the SarA consensus binding site [56] revealed two regions that contain this sequence, centered around −168 and −53 nucleotides. In addition, we identified three sites that harbor the 7-bp consensus binding sequence for the winged-helix-turn-helix DNA-binding proteins, which SarA is a member (Figure 6) [57]. The 7-bp consensus binding sequence appears in pairs in P*fmtA*; two pairs are within the 26-bp SarA binding consensus sequences and the third pair is centered on the −112 nucleotide (Figure 6). The EMSA studies with DNA fragments derived from P*fmtA*, harboring the 26-bp (*seqA1*) or the 7-bp SarA binding sequences (*seqA2*), showed that SarA is recruited to these sites. The DNA fragment that did not contain any of these sequences (*seqB1*) failed to recruit SarA, suggesting that SarA recognize specific sequences in P*fmtA*, known to interact with SarA, and that it may utilize both these sites to bind to the *fmtA* promoter.

SarA is a global transcriptional regulatory protein linked to the regulation of numerous virulence factors [58]–[60]. It is a 124-residue protein that forms dimers in solution and binds as a dimer to the target promoters. SarA also belongs to the winged helix-turn-helix family of proteins. Several *S. aureus* proteins share high sequence similarities with SarA and are grouped into a SarA family of proteins [44].

Based on the structural information on the SarA family of proteins, four regulatory mechanisms are proposed for SarA [44]: 1) bending of the target DNA to facilitate contact with the regulatory proteins; 2) the formation of three SarA dimers which hold the DNA in a closed configuration not amenable to transcription; 3) the formation of a heterodimer between compatible family members that may interfere with the function of the homodimer and 4) competitive displacement of one homolog by another. In the case of the *fmtA* promoter, it is possible that SarA may follow the first regulatory mechanism whereby SarA could bind as a dimer at the three identified sites.

The SarA family of proteins has been recently linked to *S. aureus* autolysis, biofilm formation, and resistance to cell wall inhibitors [61]–[65]. A study by McAleese et al. showed that a clinical isolate of *S. aureus* with intermediate resistance levels to vancomycin (a VISA strain) exhibited higher *sarA* expression levels than the vancomycin susceptible parent strain when grown in an antibiotic-free medium [27]. The expression levels of *fmtA* were also increased in the VISA strain. Interestingly, the *sarA* levels in susceptible or methicillin-resistant strains are not reported to increase in the presence of cell wall inhibitors; our data show the same phenomenon. By contrast, *fmtA* expression increases in the presence of cell wall inhibitors despite the strain background [27], [28], [30], [52]. The question that arises is, how does SarA regulate the expression of *fmtA* when its protein levels are not altered in response to cell wall stress? It has been hypothesized that the pleiotropic regulatory capabilities of SarA could be due to posttranslational modifications [66]. Recently, it was reported that SarA is a phosphorylation target by Stk1 Ser/Thr kinase [67]. Perhaps regulation of *fmtA* in response to stress is mediated by phosphorylated SarA or other posttranslational modifications. However, we cannot exclude that another transcription factor may be involved in the regulation of *fmtA* during antibiotic-induced cell wall stress.

The *fmtA* gene is reported to be part of the VraSR regulon [55]. However, VraR was not isolated from our protocol. There could be two reasons for this: i) VraR is not involved in regulation of *fmtA* or ii) our screening protocol failed to identify VraR due to low binding affinity of VraR for the *fmtA* promoter. We have previously shown that VraR binding affinity to its own promoter is 1 µM, which is one order of magnitude higher than that of SarA for P*fmtA* [68]. It is possible that under our screening conditions the occupancy of P*fmtA* by VraR is low. A recent study by Sengupta et al. used chromatin immunoprecipitation techniques to investigate the promoters under the direct control of VraR [69]. Interestingly, VraR was not identified as the transcription factor for *fmtA*, but the study showed that other members of the VraSR regulon, such as *pbp2*, *murZ*, and *sgtB*, are under the direct regulation by VraR.

In conclusion, this study links the global regulator SarA with a penicillin-binding protein that is also involved in autolysis and biofilm formation. Our findings further extend the multiple functions of SarA and establish a link between processes involved with *S. aureus* pathogenicity, i.e., virulence factor expression and the cell wall stress response.
